# Total Intravenous Anesthesia Protocol for Decreasing Unacceptable Movements during Cerebral Aneurysm Clipping with Motor-Evoked Potential Monitoring: A Historical Control Study and Meta-Analysis

**DOI:** 10.3390/jpm13081266

**Published:** 2023-08-16

**Authors:** Yong-Seok Park, Yong-Seo Koo, Seungil Ha, Sangho Lee, Ji-Hoon Sim, Joung Uk Kim

**Affiliations:** 1 Department of Anesthesiology and Pain Medicine, Asan Medical Center, University of Ulsan College of Medicine, Seoul 05505, Republic of Korea; yongparkanesth@gmail.com (Y.-S.P.);; 2 Department of Neurology, Asan Medical Center, University of Ulsan College of Medicine, Seoul 05505, Republic of Korea; 3 Department of Anesthesiology and Pain Medicine, Kyung Hee University Hospital, Kyung Hee University College of Medicine, Seoul 02447, Republic of Korea

**Keywords:** cerebral aneurysm, motor-evoked potential, total intravenous anesthesia

## Abstract

Injury can occur during intraoperative transcranial motor-evoked potential (MEP) monitoring caused by patient movement related to insufficient neuromuscular blocking agent use. Here, we evaluated the incidence of unacceptable movements in patients undergoing intraoperative MEP monitoring following our anesthetic protocol. We reviewed the anesthesia records of 419 patients who underwent unruptured cerebral aneurysm clipping with intraoperative MEP monitoring. The anesthetic protocol included target-controlled infusion with a fixed effect-site propofol concentration of 3 μg/mL and an adjustable effect-site remifentanil concentration of 10–12 ng/mL. We compared our findings of the intraoperative parameters and incidence of spontaneous movement and respiration with those of published meta-analysis studies. Spontaneous movement and respiration occurred in one (0.2%) patient each. The meta-analysis included six studies. The pooled proportions of spontaneous movement and respiration were 6.9% (95% confidence interval [CI], 1.3–16.5%) and 4.1% (95% CI, 0.5–14.1%), respectively. The proportion of spontaneous movement in our study was significantly lower than that in previous studies (*p* = 0.013), with no significant difference in spontaneous respiration (*p* = 0.097). Following our center’s anesthesia protocol during cerebral aneurysm clipping resulted in a low incidence of spontaneous respiration and movement, indicating its safety for patients undergoing intraoperative MEP monitoring.

## 1. Introduction

Intraoperative transcranial motor-evoked potential (MEP) monitoring is used to detect and prevent neurologic injury during various surgeries, including tumor resection near the motor cortex or corticospinal tract, cerebral aneurysm clipping, spinal cord surgery, and spinal deformity correction [1,2,3]. During cerebral aneurysm clipping, motor impairment caused by cerebral artery obliteration, which cannot be sensitively detected via somatosensory-evoked potential monitoring, can be rapidly detected via intraoperative MEP monitoring [4,5]. The real-time detection of and rapid response to intraoperative neurologic injury enables the prediction and prevention of postoperative neurologic complications [5,6]. Currently, intraoperative MEP is considered valuable for neurological monitoring during cerebral aneurysm clipping. However, there are some safety concerns, including the risk of seizure, tongue or endotracheal tube biting, brain damage, or injury caused by abrupt patient movement [7,8,9].

The lack of or partial use of neuromuscular blocking agents (NMBAs) during MEP monitoring to ensure the test’s accuracy [10,11] increases the risk of intraoperative spontaneous movement. The incidence of spontaneous movement reportedly varies from 3.2% to 28.1% in patients undergoing intraoperative MEP monitoring depending on the anesthesia method or type of operation [10,12,13]. Even a single sudden movement can cause fatal outcomes, especially during cerebral aneurysm clipping, in which vital intracranial vessels are microscopically manipulated. Total intravenous anesthesia (TIVA) is generally preferred for intraoperative MEP monitoring [4]; however, few guidelines or studies mention the optimal anesthesia protocol for preventing patient movement during cerebral aneurysm clipping. For surgeries other than cerebral aneurysm clipping, the 2019 TIVA guidelines from the Association of Anaesthetists and the Society for Intravenous Anaesthesia recommend an effect-site propofol concentration of 2.5–4.0 mcg/mL and an effect-site remifentanil concentration of 2–6 ng/mL with an appropriate dose of NMBA [14]. A deeper anesthetic level would be needed during cerebral aneurysm clipping if the appropriate NMBA dose is not administered; however, propofol reduces the MEP amplitude in a dose-dependent manner [15], whereas remifentanil has minimal effects on the MEP [16].

We introduced an anesthesia protocol for cerebral aneurysm clipping with intraoperative MEP monitoring at our center and evaluated the incidence of spontaneous movement and respiration. We also explored the appropriateness of this protocol in patients undergoing MEP monitoring by conducting a literature review.

## 2. Materials and Methods

### 2.1. Study Population and Data Acquisition

This study was approved by the Institutional Review Board of Asan Medical Center (IRB No. 2018-1520, date of approval: 14 December 2018) and the requirement for written informed consent was waived due to the retrospective nature of the study. We retrospectively reviewed the electronic medical records of consecutive patients who underwent the elective microsurgical clipping of unruptured cerebral aneurysms with intraoperative MEP monitoring at our center between March 2017 and February 2018. We excluded patients with a poor general condition (American Society of Anesthesiologists Physical Status >3) and those with any additional intracranial pathologies or preoperative neurologic deficits to minimize the effects of confounding factors.

We collected baseline characteristic data for all patients, including sex, age, weight, height, underlying diseases, and laboratory findings and preoperative and intraoperative variables from the electronic medical records. Intraoperative variables included the total infused doses of propofol and remifentanil, amount of fluid administered, urine output, estimated blood loss, operation time, anesthesia time, MEP stimulus intensity, and incidence of spontaneous movement and spontaneous respiration. Spontaneous movement was defined as the interruption of the operation due to patient movement or observation of patient movement by the anesthesiologist or surgeon, and spontaneous respiration was defined as the detection of a notch in the end-tidal capnogram or observation of self-respiration by the anesthesiologist. Postoperative variables included altered mental status, motor weakness, sensory deficit, and other neurologic complications.

### 2.2. Anesthesia Protocol

After routine monitoring including electrocardiography, pulse oximetry, and non-invasive blood pressure measurement, 25–50 mg of pethidine was administered intravenously. Following local infiltration with 1% lidocaine to minimize pain during catheterization, we placed an arterial catheter for continuous blood pressure monitoring and injected 20–40 mg of intravenous lidocaine to prevent pain during propofol administration. Anesthesia was induced using 2 mg/kg of intravenous propofol. After the patient lost consciousness, we calibrated the train-of-four (TOF) monitor and injected 0.8–1 mg/kg of intravenous rocuronium to achieve neuromuscular blockade (NMB). Subsequently, we injected propofol and remifentanil using a target-controlled injection pump (Orchestra, Fresenius Vial, Brezins, France), increasing the effect-site concentration to 2 mcg/mL and 10 ng/mL, respectively, over 3 min. We then gently intubated the patient to prevent any abrupt increase in blood pressure and administered 10–30 mg of intravenous esmolol, as required. After intubation, we reduced the effect-site concentration of remifentanil to 8 ng/mL. The patient was ventilated with an air/oxygen mixture (fraction of inspired oxygen, 0.5), and we adjusted the ventilation to achieve an arterial carbon dioxide pressure of 35 ± 2 mmHg. We used Bispectral Index (Aspect Medical Systems Inc., Framingham, MA, USA) to evaluate the depth of anesthesia (target index, 40 ± 5). As the operation proceeded, we gradually increased the propofol and remifentanil concentrations to 2.5–3.0 μg/mL and 10–12 ng/mL, respectively, over 30 min. After dural opening, we set the effect-site propofol concentration to 3.0 μg/mL. As hypotension or bradycardia can occur due to propofol and remifentanil administration, we continuously infused phenylephrine (0.5–3.0 mg/h) and/or administered a bolus dose of atropine (0.25–0.5 mg) to maintain the heart rate at between 45 and 60 beats per minute, as necessary. Other than the first dose of rocuronium injected for anesthesia induction, we did not administer NMBAs intraoperatively unless it was necessary due to spontaneous movement or respiration. We noted every event, including hemodynamic change, spontaneous movement, or spontaneous respiration, in the anesthesia record.

### 2.3. MEP Monitoring and Evaluation

After inducing anesthesia and positioning the patient, we placed the MEP electrodes. We used Neuropack MEB-2200 (Nihon Kohden, Tokyo, Japan) and Digitimer Multi-Pulse Cortical Stimulator model-D185 (Digi-timer Ltd., Hertfordshire, UK) to record MEP waves. For left and right hemispheric stimulation, we placed subcutaneous electrodes at C3/C4. We delivered stimuli as a train of six constant-current, anodal, 0.5-ms-wide waves with 3 ms interstimulus intervals. We calibrated the stimulus intensity in such a way that all evoked potential responses were detected in the lower extremities. Using patch electrodes, we recorded the MEPs of the tibialis anterior, adductor hallucis, and abductor pollicis brevis muscles. We recorded the MEPs at dural opening, before vessel occlusion, during clip placement, and at dural closing. A change in MEP was defined as a decrease in MEP amplitude of >50% or loss of MEP signal in 3 consecutive trials [17]. We reviewed and described all cases of MEP changes or postoperative neurologic deficits.

### 2.4. Meta-Analysis

The patients, interventions, outcomes, and study design criteria were applied. We included studies with: (1) “patients” who underwent evoked-potential monitoring during neurosurgery, (2) general anesthesia and neuromuscular monitoring as the “intervention”, (3) no relevant “comparator”, (4) incidence of spontaneous movement as the “outcome”, and (5) original article as the “article type”. We searched the PubMed, Embase, and Cochrane Library clinical trial databases from their inception to 20 November 2022, using the following search terms to identify studies: (aneurysm OR crani* OR nerv* OR evoked OR motor) AND (neurophysi* OR train of four OR propofol OR remifentanil OR sevoflurane OR desflurane) AND movement AND (incidence OR facial nerve). We also searched the reference lists of the retrieved articles for additional relevant studies. Two reviewers independently performed the literature search and selection. Disagreements among reviewers were resolved through discussion. We applied the following exclusion criteria: (1) non-original articles such as reviews, case reports, letters, and abstracts; (2) studies from outside the field of interest; (3) studies from outside the field of neurosurgery; (4) studies not investigating TIVA; (5) sample size of <10; (6) overlap in the study population; and (7) insufficient information on study outcomes. We included the most recently published study when we encountered studies where the populations overlapped. In each study, we only included patients who underwent TIVA and patients who did not receive or received minimal NMB in the meta-analysis.

### 2.5. Statistical Analysis

Baseline characteristics, preoperative variables, and intraoperative variables have been reported using descriptive statistics. Continuous variables have been reported as the mean ± standard deviation (SD) or median (interquartile range (IQR)). Categorical variables have been expressed as frequencies or proportions. To analyze relationships after pooling the outcomes of the included studies, we calculated proportions with 95% confidence intervals (CIs). We used Clopper–Pearson confidence intervals for individual studies. The proportions were pooled through meta-analysis with a random-effects model (DerSimonian–Laird method) using the Freeman–Tukey double arcsine method for computing weights. We used the Cochran Q test and Higgins I^2^ test to assess heterogeneity among the results of different studies. We assessed the difference between our data and that of the studies in the meta-analysis using the Q test with a random-effects model applied within subgroups [18]. The sample size was the number of patients enrolled during the study period. All statistical analyses were performed using R version 4.1.3 (R Foundation for Statistical Computing, Vienna, Austria).

## 3. Results

### 3.1. Intraoperative and Postoperative Data of Enrolled Patients

We reviewed the medical records of 419 patients with no preoperative neurologic deficits who underwent unruptured cerebral aneurysm clipping and collected data on their intraoperative MEP changes and postoperative neurologic deficits. The baseline characteristics and preoperative variables of the enrolled patients are presented in Table 1. Two patients exhibited spontaneous respiration or movement (0.5%); in one of them, we observed spontaneous finger movement when the aneurysm was accessed approximately 2 h after skin incision, whereas, in the other patient, we noted notches on the capnogram, which indicated spontaneous breathing, at approximately 100 min after skin incision. In response, we administered additional NMBAs to these two patients. The total infused propofol and remifentanil doses were 1384 ± 408 mg and 5.7 ± 1.6 mg, respectively. The rates of phenylephrine and atropine administration were 93.3% and 31.2%, respectively (Table 1).

Seven patients exhibited intraoperative MEP changes (Table 2). Among them, the MEP changes in four patients were reversible, and the patients recovered after the necessary actions were performed (e.g., hemodynamic stabilization, decompression, or clip repositioning). However, the changes were not reversed until the end of the surgery in three patients. Two of these patients were administered additional NMBAs, which might have caused bilateral MEP loss. The third patient was not administered additional NMBA, and we observed a 50% decrease in unilateral MEP after permanent clipping, which did not recover until the end of the surgery despite our performing the needed actions. Nevertheless, the patient had no postoperative neurologic deficit; therefore, we considered this to be a false-positive case. The characteristics of the patients who had postoperative neurologic deficits are presented in Table 2. Five patients had postoperative neurologic deficits, such as altered mental status or hemiparesis, caused by cerebral infarction, subdural hemorrhage, or intracerebral hemorrhage. In most patients, the symptoms resolved, resulting in normal neurologic functioning or minimal sequelae. However, the patient who developed a coma following intracerebral hemorrhage did not recover.

### 3.2. Meta-Analysis of the Effect of TIVA on Spontaneous Movement and Respiration during Neurosurgery

**Figure 1:**
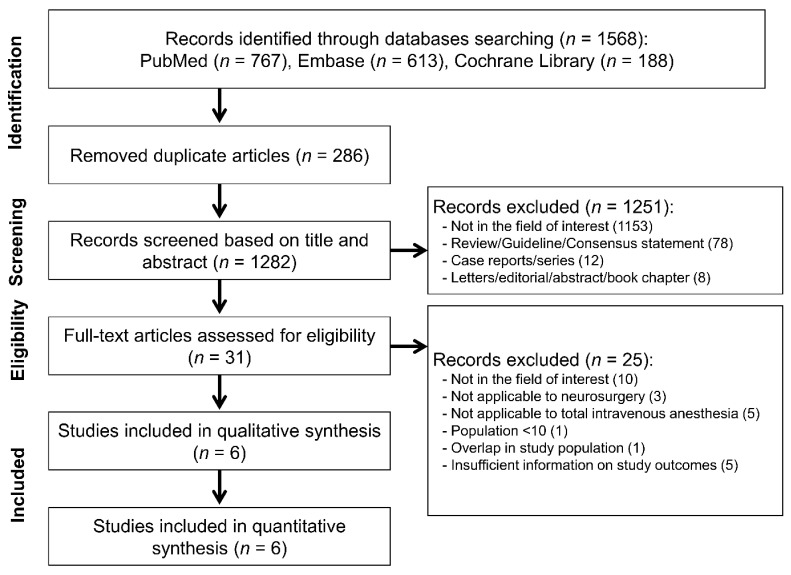
Flow chart of study selection for the meta-analysis.

Our electronic search yielded 1568 articles, including 767 from PubMed, 613 from Embase, and 188 from Cochrane Library (Figure 1). Of these, 31 articles were considered potentially eligible. After full-text review, we excluded 25 articles for the following reasons: not from the field of interest (n = 10), not involving neurosurgery (n = 3), not investigating TIVA (n = 5), sample size of <10 (n = 1), overlap in the study population (n = 1), and insufficient information on study outcomes (n = 5). Finally, six studies were included in the meta-analysis (Figure 1).

Table 3 summarizes the anesthesia protocols and outcomes of the included studies. A total of 637 patients underwent evoked potential monitoring during neurosurgery under general anesthesia with TIVA in the studies. In the study by Jellish et al. [13], the patients in the TIVA group received a continuous infusion of propofol at 100–200 μg/kg/min and remifentanil at 0.25–0.5 μg/kg/min, and the incidence of movement events was 3.2% (1/31). In the study by Hemmer et al. [12], anesthesia was usually maintained using ≤150 μg/kg/min of propofol, ≥0.1 μg/kg/min of remifentanil, and ≤0.5 minimum alveolar concentration of desflurane, and 7 (3.2%) of the 220 patients exhibited unacceptable movements. In the study by Kim et al. [10], propofol (effect-site concentration, 3–6 μg/mL) and remifentanil (2–5 ng/mL) were infused via a target-controlled infusion (TCI) pump for anesthesia maintenance. The incidence of spontaneous movement and respiration was 3.3% (1/30) and 3.3% (1/30), respectively, in the no NMB group. Kim et al. [17] evaluated the diagnostic accuracy of MEP monitoring during cerebral aneurysm clipping. In their study, no spontaneous movement or spontaneous breathing events were reported in any of the 276 patients who underwent aneurysm clipping with no NMB. In the study by Ko et al. [19], propofol (effect-site concentration, 2–5 μg/mL) and remifentanil (2–4 ng/mL) were infused via a TCI pump for anesthesia maintenance. The incidence of spontaneous movement and respiration was 30.0% (12/40) and 15.0% (6/40), respectively. In the study by Zhang et al. [20], anesthesia was maintained using a continuous infusion of propofol (100–150 μg/kg/min) and remifentanil (0.1–0.3 μg/kg/min). The incidence of spontaneous movement and respiration was 15.0% (6/40), and 2.5% (1/40), respectively, in the group receiving continuous infusion of muscle relaxants at 6.0 mcg/kg/min.

**Figure 2:**
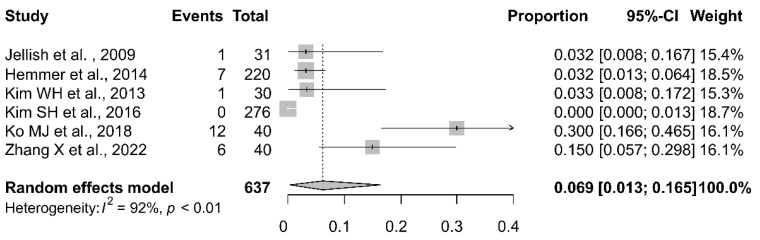
Forest plot of intraoperative spontaneous movement during motor-evoked potential monitoring in patients undergoing neurosurgery. CI, confidence interval, [10,12,13,17,19,20].

**Figure 3:**
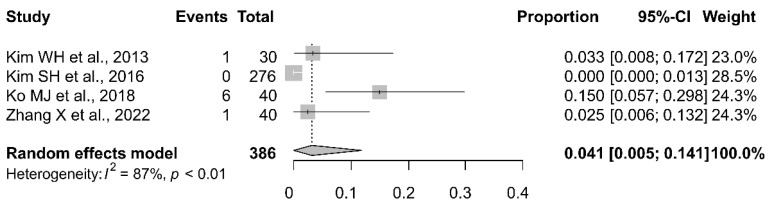
Forest plot of intraoperative spontaneous respiration during motor-evoked potential monitoring in patients undergoing neurosurgery, [10,17,19,20].

The meta-analytic pooling of the findings of all six studies that investigated intraoperative spontaneous movement revealed a pooled proportion of 6.9% (95% CI, 1.3–16.5%) (Figure 2). The Higgins I^2^ statistics demonstrated substantial heterogeneity (I^2^ = 92%). The comparison between the findings of the present study and those of the six previous studies revealed that the proportion of spontaneous movement in the present study (proportion, 0.2%; 95% CI, 0.0–1.3%) was significantly lower than that in the previous studies (*p* = 0.013). The meta-analytic pooling of the findings of all four studies that investigated intraoperative spontaneous respiration revealed a pooled proportion of 4.1% (95% CI, 0.5–14.1%) (Figure 3). The Higgins I^2^ statistics demonstrated substantial heterogeneity (I^2^ = 87%). The comparison between the findings of the present study and those of the four previous studies revealed that the proportion of spontaneous respiration in the present study (proportional, 0.2%; 95% CI, 0.0–1.3%) was lower than that in the previous studies, although this difference was not statistically significant (*p* = 0.097).

## 4. Discussion

Our study showed that intraoperative events could be detected using intraoperative MEP monitoring. Here, the incidence of spontaneous respiration or movement was 0.2%. In the meta-analysis of the effect of TIVA on spontaneous movement and respiration during neurosurgery, the pooled proportions of spontaneous movement and respiration were 6.9% and 4.1%, respectively. The comparison between the findings of the present study and those of previous studies revealed that the proportion of spontaneous movement in the present study was significantly lower than that in the previous studies; this was also noted for the proportion of spontaneous respiration, although this difference was not statistically significant. These results showed that the TIVA protocol used at our center allows for appropriate intraoperative MEP monitoring while reducing unacceptable movements during cerebral aneurysm clipping.

Our study differs from other studies in the anesthesia protocol and concentrations of propofol and remifentanil used. While TIVA using TCI was performed at our center, various anesthetic methods, including inhaled anesthesia, intravenous anesthesia with continuous infusion, and combined methods, were used in the other studies. Compared with the TCI protocol used in one of the reviewed reports [10], our anesthesia protocol targeted a relatively higher effect-site remifentanil concentration (10–12 ng/mL vs. 2–5 ng/mL) and a relatively lower and fixed effect-site propofol concentration (3 μg/mL vs. 3–6 μg/mL) to maintain anesthesia. Other studies, in which inhaled and intravenous anesthetics were used in combination, reported varying ranges for the incidence of spontaneous respiration and movement [12,13,21]. Given that the probability of movement in response to surgical stimuli decreases as the remifentanil infusion rate increases [21], we adjusted the effect-site remifentanil concentration to suppress patient movement, while maintaining a steady effect-site propofol concentration to minimize MEP reduction. Thus, unlike the protocols used in previous studies, our anesthesia protocol was characterized by our targeting a deep anesthetic depth by fixing the propofol concentration and adjusting the remifentanil concentration to >10 ng/mL.

The higher effect-site concentration of remifentanil might be a factor driving the lower incidence of patient movement in this study compared with previous studies. Maurtua et al. [21] investigated the incidence of movement in patients undergoing cerebral aneurysm clipping at 12 different remifentanil doses and found that the incidence of movement substantially decreased as the remifentanil infusion rate increased (probability of movement, 65.4% at 0.10 μg/kg/min and 20.7% at 0.21 μg/kg/min). In another study comparing the effects of propofol and remifentanil on sedation and movement prevention during periretrobulbar block, the remifentanil group showed a lower incidence of patient movement while having similar hemodynamic and respiratory parameters as the propofol group [22]. These results are consistent with ours in that they demonstrate the effect of remifentanil on preventing movement. In particular, the dose-dependent decrease in the probability of patient movement in the study of Maurtua et al. might explain the low incidence of patient movement achieved using our center’s protocol, where the effect-site remifentanil concentration was kept relatively high. Considering that increasing the remifentanil concentration markedly reduces the minimum alveolar concentration and prevents movement in 50% of patients [23], we can infer that a high remifentanil concentration induces deep anesthesia along with TIVA, thereby preventing movement.

In addition to deep anesthesia, the effect of glycine might have prevented movement. Clinical formulations of remifentanil (Ultiva^®^) contain 15 mg of glycine as an acidic buffer, and glycine can act as an inhibitory neurotransmitter and cause reversible motor weakness when injected intrathecally in rodents [24]. The accidental administration of Ultiva^®^ into the epidural space instead of local anesthetics for pain control reportedly caused reversible respiratory depression [25,26]. As glycine receptors play a role in the immobilization produced by inhaled anesthetics [27], the systemic administration of Ultiva^®^ may contribute to immobilization during anesthesia. Furthermore, intravenous glycine administration reportedly increases serum and cerebrospinal fluid (CSF) glycine concentrations in a dose-dependent manner [28], with an increase in the CSF glycine concentration from 9.5 μmol/L to 15 μmol/L being reported after remifentanil infusion for sedation in the intensive care unit [29]. Zhang et al. suggested that glycine receptors, which are predominantly located in the spinal cord, are more relevant mediators of immobility in response to ascending noxious stimuli than gamma-aminobutyric acid A receptors [27,30]. Sumie et al. further demonstrated that the glycine in Ultiva^®^ hyperpolarizes the postsynaptic membrane in substantia gelatinosa neurons by activating chloride channels and suppressing excitatory postsynaptic currents through its receptors [31]. Glycine modulates nociception from ascending noxious stimuli by activating its postsynaptic and presynaptic receptors. Therefore, a higher effect-site remifentanil concentration may influence immobility during general anesthesia in the absence of muscle relaxants. The CSF glycine concentration is only 1/100 of that in the plasma because of the blood–brain barrier, which strictly limits amino acid transport into the brain [32]; however, we hypothesize that an elevated intravenous Ultiva^®^ concentration would increase the CSF glycine concentration and may also affect pain perception or decrease movement.

One possible benefit of maintaining a fixed effect-site propofol concentration is that it enables the minimization of propofol-induced MEP amplitude suppression. As propofol reduces MEP amplitude in a dose-dependent manner [15,33,34], maintaining a fixed and relatively low propofol effect-site concentration has the advantage of allowing for MEP amplitude to be consistently maintained at a relatively low stimulus voltage. Using a low stimulus intensity may reduce false-negative MEP monitoring results and prevent harmful events such as endotracheal tube biting or excessive patient movement [10,17,35]. However, maintaining an effect-site remifentanil concentration above 10 ng/mL, as in our protocol, may induce relatively deep anesthesia and cause side effects such as bradycardia and hypotension. Therefore, when applying our protocol, the remifentanil concentration must be gradually increased, and vasopressors and anticholinergics should be administered with appropriate titration [36].

Our study has some limitations. First, the retrospective descriptive observational study design did not allow for statistically rigorous comparison with other protocols or controls. Nevertheless, we attempted to overcome this by performing a meta-analysis of similar studies for comparison. Well-designed prospective studies are needed to confirm the generalizability of our protocol. Second, as this study involved data collection via electronic medical record review, some intraoperative events or findings may have been omitted or not recorded. Third, although the TOF was monitored intraoperatively, it was not recorded or analyzed. Because the absence of NMB other than that from the induction dose is an important element of this protocol, TOF recordings would have helped to identify changes in NMB. Lastly, the somatosensory-evoked potential records were not reviewed in this study.

## 5. Conclusions

Using our anesthesia protocol, in which the effect-site propofol concentration is fixed at 3 μg/mL and that of remifentanil is adjusted to a level above 10 ng/mL, a low incidence of spontaneous movement and respiration could be achieved without additional NMB during cerebral aneurysm clipping. The results of our comparative analysis between the results of the present study and those of the meta-analysis demonstrate the applicability of our protocol.

## Figures and Tables

**Table 1:** Baseline characteristics and intraoperative data of the 419 enrolled patients.

Variables	Value
Baseline characteristics	
Male	126 (30.1)
Female	293 (69.9)
Age (years)	59.7 ± 9.3
Weight (kg)	63.6 ± 10.4
Hypertension	218 (52.0)
Diabetes mellitus	46 (11.0)
Intraoperative data	
Propofol infusion rate (mcg/kg/min)	82.0 ± 15.4
Remifentanil infusion rate (mcg/kg/min)	0.34 ± 0.07
Operation time ^†^ (min)	183.3 ± 52.0
Anesthesia time (min)	267.8 ± 61.1
Crystalloids infused (mL)	1697.2 ± 502.4
Urine output (mL)	836.5 ± 496.3
Estimated blood loss (mL)	190.2 ± 127.7
Phenylephrine administration	391 (93.3)
Atropine administration	130 (31.0)
MEP stimulus intensity (V)	267.5 ± 53.7
Spontaneous movement	1 (0.2)
Spontaneous respiration	1 (0.2)

MEP, motor-evoked potential. ^†^ Defined as the time from skin incision to skin closure. Values are expressed as n (%) or mean ± standard deviation.

**Table 2:** Overview of the 12 patients with intraoperative MEP changes and postoperative neurologic deficits from the 419 patients who underwent unruptured cerebral aneurysm clipping.

Location of Aneurysm	Location of MEP Change	Reversibility of MEP Change	Probable Reasons for MEP Change	Postoperative Neurologic Deficit	Remarks
Patients with MEP changes					
Rt PCoA	Lt APB, TA, AH	Reversible	Temporary compression due to retraction	Third CN palsy	MEP recovered after decompression
Rt PCoA	Lt APB	Reversible	Aneurysm rupture	None	MEP recovered after hemodynamic stabilization
ACoA	APB	Reversible	Permanent clipping	None	MEP recovered after clip repositioning
Rt MCA	Lt TA	Reversible	Permanent clipping	None	MEP recovered after clip repositioning
Rt PCoA	Both TA	Irreversible	NMB	None	
Lt A1, MCA	Rt TA	Irreversible	Permanent clipping	None	
Rt MCA	All	Irreversible	NMB	None	Non-compliance with the anesthesia protocol
Patients with postoperative neurologic changes					
ACoA	None			Stupor	Postoperative SDH
ACoA	None			Coma	Intraoperative ICH
Patients with perforating artery injury					
ACoA	None			Confusion	Postop ACA infarction
Lt PCoA	None			Rt hemiparesis	Postop MCA infarction
Lt MCA	None			Rt hemiparesis	Lt BG infarction

Rt, right; PCoA, posterior communicating artery; ACoA, anterior communicating artery; MCA, middle cerebral artery; Lt, left; A1, proximal segment of anterior cerebral artery; APB, abductor pollicis brevis; TA, tibialis anterior; AH, abductor hallucis; NMB, neuromuscular blockade; CN, cranial nerve; SDH, subdural hemorrhage; ICH, intracerebral hemorrhage; ACA, anterior cerebral artery; BG, basal ganglia.

**Table 3:** Anesthesia protocols and outcomes of the studies included in the meta-analysis.

Study Details	Study Design	Surgery Type	Anesthesia Protocols	Additional NMB ^†^	Spontaneous Movement	Spontaneous Respiration
Jellish et al. (2009) [13]	Randomized controlled trial	Craniofacial and skull-based surgeries	Propofol (100–200 mcg/kg/min) + Remifentanil (0.25–0.5 mcg/kg/min)	No	1/31 (3.2)	Not evaluated
Hemmer et al. (2014) [12]	Retrospective study	Craniotomy for cerebral aneurysm clipping	Desflurane (≤0.5 MAC) + Remifentanil (≤0.1 mcg/kg/min) + Propofol (0–150 mcg/kg/min)	No	7/220 (3.2)	Not evaluated
Kim et al. (2013) [10]	Randomized controlled trial	Cerebral aneurysm clipping, brain tumor resection, spinal laminectomy	Propofol (3–6 mcg/mL) + Remifentanil (2–5 ng/mL)	No	1/30 (3.3)	1/30 (3.3)
Kim et al. (2016) [17]	Retrospective study	Cerebral aneurysm clipping	Propofol (3 mcg/mL) + Remifentanil (10–15 ng/mL)	No	0/276 (0)	0/276 (0)
Ko et al. (2018)	Randomized controlled trial	Cerebral aneurysm coil embolization or stent insertion	Propofol (2–5 mcg/mL) + Remifentanil (2–4 ng/mL)	0.1 mg/kg to manage any involuntary movement or at the request of the surgeon	12/40 (30.0)	6/40 (15.0)
Zhang et al. (2022)	Randomized controlled trial	Spinal surgery	Propofol (100–150 mcg/kg/min) + Remifentanil (0.1–0.3 mcg/kg/min)	6.0 mcg/kg/min continuously infused	6/40 (15.0)	1/40 (2.5)

MAC, minimum alveolar concentration. ^†^ NMB other than that from the induction dose.

## Data Availability

The dataset used and/or analyzed during the current study is available from the corresponding author on reasonable request.
